# Flexible and Wearable Zinc-Ion Hybrid Supercapacitor Based on Double-Crosslinked Hydrogel for Self-Powered Sensor Application

**DOI:** 10.3390/ma15051767

**Published:** 2022-02-26

**Authors:** Xi Wen, Kang Jiang, Heng Zhang, Hua Huang, Linyu Yang, Zeyan Zhou, Qunhong Weng

**Affiliations:** 1 School of Physical Science and Technology, Xinjiang University, Urumqi 830046, China; www6231659@163.com; 2 College of Materials Science and Engineering, Hunan University, Changsha 110016, China; jiangkang2@hnu.edu.cn (K.J.); 15873400812@163.com (H.Z.); 3 Xinjiang Lixin Energy Co., Ltd., Urumqi 830046, China; huahuag_888@163.com

**Keywords:** hydrogel, crosslinked, Zn, supercapacitor, self-powered sensor

## Abstract

The rapidly growing Internet of Things (IoT) has brought about great demand for high-performance sensors as well as power supply devices for those sensors. In this respect, the integration of sensors and energy storage devices, or the development of multifunctional devices having both energy storage and sensing properties, is of great interest in the development of compact sensing systems. As a proof of concept, a zinc-ion hybrid supercapacitor (ZHS) based on a double-crosslinked hydrogel electrolyte is developed in this work, which can be employed not only as an energy storage device, but also as a self-powered sensor for human movement and breathing detection. The ZHS delivers a capacitance of 779 F g^−1^ and an energy density of 0.32 mWh cm^−2^ at a power density of 0.34 mW cm^−2^, as well as sensitive resistance response to strain. Our work provides a useful basis for future designs of self-powered sensing devices and function-integrated systems.

## 1. Introduction

The Internet of Things (IoT), as well as portable and human interaction electronic devices, are developing at an astonishing speed [1,2,3,4]. As a result, the demand for high-performance, highly-secure and highly-flexible energy storage devices is increasing rapidly to meet growing power supply requirements. Among various energy storage devices, zinc-ion hybrid supercapacitors (ZHS) are beginning to receive extensive attention due to their good electrochemical performance, low cost, high safety and environmental friendliness [5,6,7,8]. A typical sandwich-configured ZHS device comprises a cathode and an anode, together with an electrolyte layer. These parts contribute significantly to the electrochemical performances of the ZHS.

For a ZHS, the electrolyte material determines the ion transportation efficiency and the voltage window [9,10], which directly influence the performance of the device as well as the introduction of additional functions. In recent years, hydrogels made of elastic cross-linked hydrated polymer chains have been considered as electrolyte materials for zinc-ion energy storage devices due to their unique mechanical and electrical properties [11]. Hydrogels have been widely applied for electronic skin [12,13,14,15], sensors [16,17,18,19,20], wearable devices [21,22,23,24], etc. For zinc-ion energy storage electrolyte applications, they have also demonstrated excellent performance in terms of improving device energy density and the voltage window. For example, Yang et al. reported a ZHS comprising solid cellulose hydrogel as an electrolyte, which exhibited a capacity of 347 F g^−1^ and an energy density of 192 Wh kg^−1^ at a power density of 16,976 W kg^−1^ [25]. Liu et al. designed a gel electrolyte based on a polyvinyl alcohol (PVA)/Zn/ethylene glycol system. This electrolyte exhibited an ionic conductivity of 15.03 mS cm^−1^ at room temperature and maintained good ionic conductivity properties at low temperatures (9.05 mS cm^−1^ at −20 °C and 3.53 mS cm^−1^ at −40 °C) [26]. Moreover, through a solid-state zwitterionic hydrogel electrolyte design, Lu et al. fabricated the ZHS with a voltage window of 2.4 V and a maximum energy density of 286.6 Wh kg^−1^ at a power density of 220 W kg^−1^ [27]. These interesting results suggest the great potential of the hydrogel-based ZHSs as advanced power sources.

Conventional sensors require external power supply devices, which limits their potential application in dispersive application scenarios. Therefore, the development of self-powered sensors that have both sensing and energy storage functions may provide a solution to this problem. As a proof of concept, in this work, we designed a bifunctional zinc-ion hybrid supercapacitor with both high-performance energy storage and sensing properties by introducing a double-crosslinked hydrogel as the solid electrolyte. The sandwich-configurated device employs vanadium nitride (VN) and zinc foils as the cathode and anode materials, respectively, and Zn-alginate/PAAm (Polyacrylamide) hydrogel as the solid electrolyte. Through the double-crosslinked hydrogel design, the toughness of the supercapacitor is remarkably enhanced and the resistance sensing property can be exploited via external mechanical triggering. As a result, the assembled ZHS has a voltage window of 1.3 V and exhibits a capacitance of 779 F g^−1^ and energy density of 0.32 mWh cm^−2^ at a power density of 0.34 mW cm^−2^. In addition, the ZHS fabricated based on the Zn-alginate/PAAm hydrogel exhibits sensitive resistance response to body movements, breathing, etc. This concept provides an alternative and effective solution for the development of compact function-integrated devices.

## 2. Materials and Methods

### 2.1. Materials

Acrylamide (Analytical Reagent, AR, 99%) was purchased from Maclean, ShangHai, China. *N*,*N*′-methylenebisacrylamide (99%), sodium alginate (200 ± 20 mpa.s) and zinc sulfate heptahydrate (AR) were purchased from Aladdin, Los Angeles, CA, USA; ammonium persulfate (AR, 98.5%), melamine (AR) and ammonium metavanadate (AR, 99%) were purchased from Rhawn, ShangHai, China. Zinc flakes (99%) were purchased from Gaoke New Materials, Co., Ltd., GuangZhou, China.

### 2.2. Synthesis of VN

First, 2.8 mmol ammonium metavanadate and 43 mmol melamine were dissolved in 60 mL DI water, and stirred vigorously for 8 h at room temperature. Then, the precursors were collected by filtering and drying at 60 °C for 24 h. Subsequently, after grinding with a mortar, the precursor powder was placed in a tube furnace and annealed at 900 °C for 2 h in a N_2_ atmosphere.

### 2.3. Synthesis of Zn-Alginate/PAAm Hydrogels

First, 84.0 mmol acrylamide, 0.023 mmol *N*,*N*′-methylenebisacrylamide and 0.11 mmol ammonium persulfate were added to 40 mL DI water and stirred for about 30 min. Then, 2.8 mmol sodium alginate was added. The mixture was stirred until a homogeneous solution formed. The bubbles in the gel solution were removed by a vacuum pump. Then, the gel solution was poured into a specific mold for crosslinking reactions at high temperature to obtain the Na-alginate/PAAm hydrogel. Finally, the hydrogel was immersed into ZnSO_4_ aqueous solution for 2 h to perform ion exchanges and additional crosslinking reactions via the Zn^2+^ coordination, ultimately yielding the robust Zn-alginate/PAAm hydrogel.

### 2.4. Structural Characterizations

The morphology and composition of the aforementioned materials were characterized by scanning electron microscopy (SEM, Tescan MIRA3 LMH, Brno, Czech Republic) and X-ray diffractometer (XRD, Bruker D8 Advance, Karlsruhe, Germany). The tensile properties were measured on a universal material testing machine (AG-X plus, Shimadzu Corporation, Shimane, Japan) at a deformation rate of 50 mm min^−1^ at room temperature.

### 2.5. Electrochemical Characterizations

VN, acetylene black and polyvinylidene fluoride (PVDF) were dispersed in NMP at a ratio of 7:2:1. The slurry was consequently coated on carbon paper (1.0 × 2.0 cm^2^) and dried as the cathode. Then, the ZHS device was assembled by stacking the cathode sheet, hydrogel sheet and Zn sheet into a sandwich-like structure. Typically, the active material loaded on the electrode was 2.5 mg cm^−2^. Cyclic voltammetry (CV) and constant current charge-discharge (GCD) measurements were performed on an electrochemical workstation (CORRTEST CS235OH) for electrochemical performance evaluations. The cycling stability measurement of the ZHS was carried out on a LAND test system (CT2001A) with a sweeping charge and discharge rate at 2.0 A g^−1^ for 1000 consecutive cycles. The resistance changes of the self-power sensor in a different state were obtained by electrochemical workstation (CORRTEST CS235OH).

## 3. Results and Discussion

### 3.1. Synthesis of Zn-Alginate/PAAm Hydrogel Electrolyte

**Figure 1:**
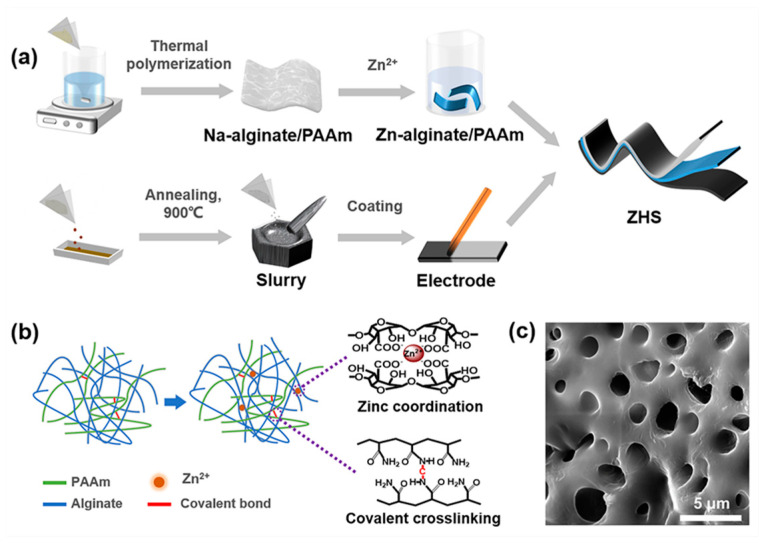
Preparation of Zn-alginate/PAAm hydrogel and assembly of ZHS device. (**a**) Illustration of fabrication process of ZHS. (**b**) Illustration of the synthesis of double-crosslinked Zn-alginate/PAAm hydrogel. (**c**) SEM image of Zn-alginate/PAAm hydrogel.

As schematically illustrated in Figure 1a, the Zn-alginate/PAAm hydrogel was synthesized through double-crosslinking procedures and assembled into the ZHS device with a sandwich-like structure. The Zn-alginate/PAAm hydrogel was synthesized in a two-step process. Firstly, alginate was added to the acrylamide monomer solution to prepare the alginate/PAAm hydrogel. As illustrated in Figure 1b, the PAAm and the sodium alginate chains were covalently crosslinked through *N*,*N*′-methylenebisacrylamide crosslinkers. Then, the alginate/PAAm hydrogel was immersed in 1.5 M ZnSO_4_ solution for ion exchange and additional Zn-ion coordination. An SEM image of the hydrogel revealed a large number of pores in the hydrogel structure, which could be used to store ZnSO_4_ solution (Figure 1c). Moreover, the intake of ZnSO_4_ aqueous solution provided working Zn ions for the ZHS and improved the ion conductivity of the hydrogel, which are both important for boosting the electrochemical performance of the fabricated ZHS.

### 3.2. Mechanical Properties of Zn-Alginate/PAAm Hydrogel

**Figure 2:**
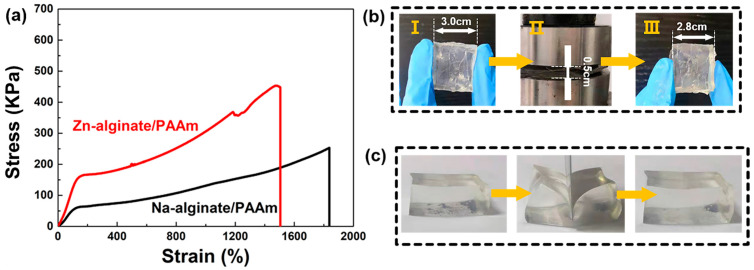
Mechanical properties of Zn-alginate/PAAm hydrogel. (**a**) Stress-strain curves of the hydrogels with and without Zn^2+^ coordination. The inset is the stretching test of Zn-alginate/PAAm hydrogel. (**b**) Photographs of Zn-alginate/PAAm hydrogel for compressing tests. (**c**) Photographs of cutting resistive test for a 10 mm-thick Zn-alginate/PAAm hydrogel.

As shown in Figure S1, the prepared Zn-alginate/PAAm hydrogel was colorless, transparent, extremely flexible and viscous, and could adhere to various types of surfaces. Furthermore, a strip of the Zn-alginate/PAAm hydrogel with 20 × 2 × 0.3 cm could support a 600 g-weight metal without breaking, proving the excellent mechanical strength of the material (Figure S2a). Under the action of external force, the stretching amount of the hydrogel could reach 1500% (Figure S2b). These impressive tensile properties may be attributed to the dynamic coordination of Zn^2+^ with the alginate molecular chain [28]. Details of the tensile tests of the hydrogels are shown in Figure 2a. As a comparison, the strain of the hydrogels after the coordination of Zn^2+^ was 1500%, i.e., lower than that of the hydrogel without Zn^2+^. The tensile strength almost doubled (460 kPa vs. 240 kPa) and the elastic modulus enhanced significantly (108 kPa vs. 47 kPa) for the Zn-alginate/PAAm hydrogel. These results suggest that the synthesized Zn-alginate/PAAm hydrogel would be ideal as a ZHS solid electrolyte or in sensor applications.

In addition to the tensile properties, the deformation recovery characteristics and cutting resistance properties of the Zn-alginate/PAAm hydrogel were also explored and analyzed. For common hydrogels, external force can only be dissipated through the rupture of polymer chains [29]. This may permanently damage the hydrogel. Nevertheless, for the present, double-crosslinked Zn-alginate/PAAm hydrogel, ionic crosslinks break and dissipate energy while the covalent crosslinks remain intact, and thus, the hydrogel structure is maintained. Due to this special energy dissipation mechanism [30], the Zn-alginate/PAAm hydrogel can be compressed to a height of 0.5 cm from an initial height of 3.0 cm while maintaining structural integrity. After unloading the stress, the height was restored to 2.8 cm (Figure 2b). As displayed in Figure 2c, the cutting resistance of the hydrogel was also explored. A 1.0 cm-thick Zn-alginate/PAAm hydrogel was cut using a blade. When the cutting depth was smaller than 0.8 cm, the hydrogel remained intact and returned to its initial state after about 2 min. This result suggests that the designed double-crosslinked Zn-alginate/PAAm hydrogel possesses excellent cutting resistance.

### 3.3. Electrochemical Performances of ZHS

**Figure 3:**
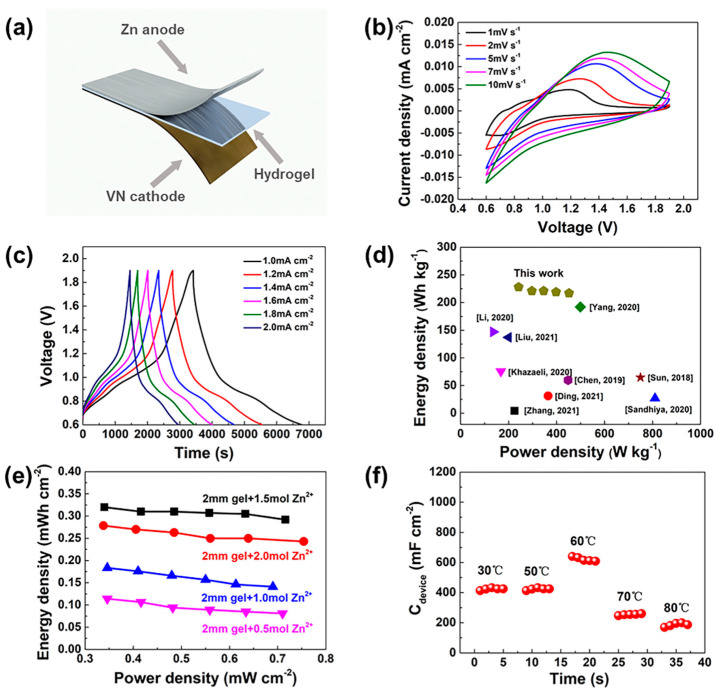
Electrochemical performances of ZHS. (**a**) Schematic illustration of sandwich-like structure of the ZHS. (**b**) CV profiles at different scanning speeds. (**c**) GCD curves at different current densities. (**d**) Comparison of energy density and power density among reported cutting-edge supercapacitors. (Yang, 2020 [25]; Liu, 2021 [26]; Chen, 2019 [33]; Zhang, 2021 [34]; Ding, 2021 [35]; Sandhiya, 2020 [36]; Khazaeli, 2020 [37]; Li, 2020 [38]; Sun, 2018 [39]) (**e**) Ragone plots of the ZHSs with the hydrogel prepared with different Zn^2+^ concentrations. (**f**) Discharge capacitance of ZHS at different temperatures.

The electrochemical performance of the ZHS was comprehensively investigated. Firstly, the water retention capacities of Zn-alginate/PAAm and Na-alginate/PAAm hydrogels were evaluated. As the results show in Figure S3, the hydrogel after Zn^2+^ coordination demonstrated enhanced water retention capacity; this characteristic is very important for solid state electrolyte applications. The whole ZHS device was assembled with a VN cathode, Zn anode and a hydrogel electrolyte layer, as shown in Figure 3a. The prepared VN (Figure S4) and acetylene black slurry were evenly coated on 0.8 mm-thick carbon paper (1 × 2 cm^2^) as the cathode. It was noted that the VN had the advantages of high conductivity [31] and a wide voltage window [32] when employed as a supercapacitor electrode material. The impedance spectrum of the ZHS is shown in Figure S5, indicating the good conductivity of the assembled device with a contact resistance as low as 13.2 Ω and a charge transfer resistance of 10.6 Ω. More specifically, the ZHS had a stable working voltage window of 0.6–1.9 V which exhibited similar shapes at different scanning speeds, as shown in Figure 3b. The galvanostatic charge-discharge curves of ZHS (Figure 3c) confirmed the ultralong discharge time at different current densities. At a current density of 1.0 mA cm^−2^, the discharge capacitance of the ZHS device is as high as 1.364 F cm^−2^, while the capacitance remained at 1.244 F cm^−2^ when the current density was further increased to 2.0 mA cm^−2^. The calculated energy density for the ZHS device was 242 Wh kg^−1^ at a power density of 228 W kg^−1^, i.e., superior to those of other sandwich-structure supercapacitors (Figure 3d) [25,26,33,34,35,36,37,38,39]. The cyclic stability test of the ZHS (Figure S6) showed 90% retention after 1000 cycles, indicating stable charge–discharge performance. The CV curves of the device at large sweep speeds were also tested (Figure S7), suggesting excellent rate performance.

The effect of the Zn^2+^ concentration on the electrochemical performance of the ZHS device was also studied. The Na-alginate/PAAm hydrogels were immersed in ZnSO_4_ solutions with different concentrations. The results of electrochemical tests showed that the Zn-alginate/PAAm hydrogel subjected to a treatment of 1.5 mol L^−1^ ZnSO_4_ solution was optimal. More specifically, for the hydrogel prepared at a Zn^2+^ concentration of 0.5 mol L^−1^, the corresponding ZHS achieved an energy density of 0.34 mWh cm^−2^ and a power density of 0.11 mW cm^−2^ at 1.0 mA cm^−2^. With a Zn^2+^ concentration reaching 1.5 mol L^−1^, the energy density significantly increased to 0.32 mWh cm^−2^ at 0.34 mW cm^−2^ (see Figure 3e).

Interestingly, the ZHS fabricated based on the double-crosslinked Zn-alginate/PAAm hydrogel showed impressive characteristics with regard to operation at high temperatures. As shown in Figure S8, after being buried in an 80 °C sand bath for 2 h, the ZHS device could still power a digital clock normally. Even in the flame of an alcohol blowtorch, the ZHS still worked for a certain period (Figure S9). We systematically studied the capacitance evolution of ZHS under different temperatures, as shown in Figure 3f. From 30 °C to 60 °C, the ZHS capacitance increases from 850 mF cm^−2^ to 1280 mF cm^−2^, while it decreases rapidly to 375 mF cm^−2^ at 80 °C. This phenomenon is likely due to the much enhanced ion diffusion rate within the hydrogel at this temperature [40]; higher temperatures lead to obvious water loss and lower ion diffusion rates, resulting in reduced capacitance. Nevertheless, at a temperature of 70 °C, the ZHS retained about 60% of its initial capacity, suggesting that the proposed device has good high-temperature performance.

In addition, the supercapacitor performance under different bending conditions was also evaluated. As the results in Figures S10 and S11 show, the ZHS retained its powering functions and high discharge capacitance, even when bent to 90°. These characteristics show that the ZHS fabricated with the Zn-alginate/PAAm hydrogel possesses excellent resistance to external impacts, and is also highly adaptable to various situations.

### 3.4. Self-Powered Sensing Properties

**Figure 4:**
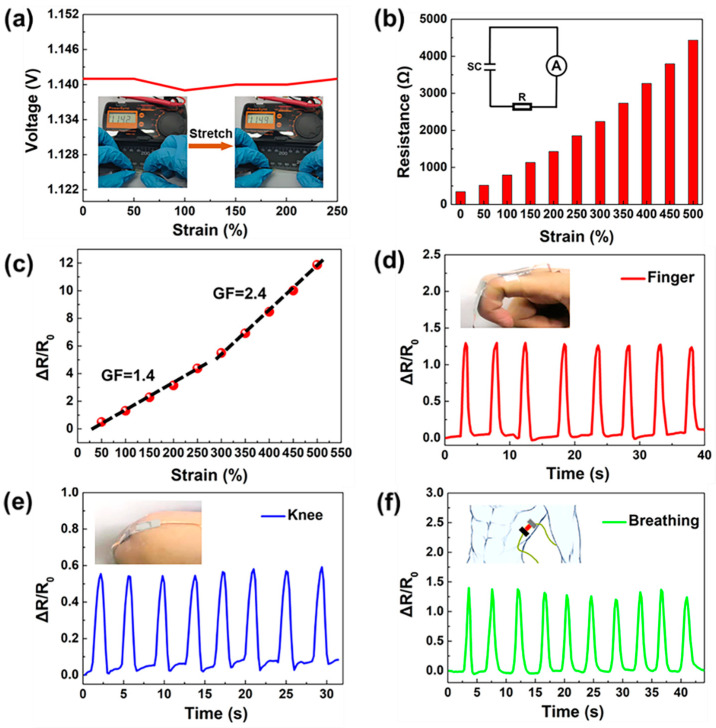
Self-powered sensing performance of the ZHS device. (**a**) Output voltage of the ZHS under strain. (**b**) Resistance response of the sensor with loaded strains from 0 to 500%. (**c**) Relative resistance change ΔR/R_0_ of the device under varying strain. (**d**) Relative resistance response ΔR/R_0_ to repeated bending by hand. (**e**) ΔR/R_0_ change along with repeated joint movements. (**f**) ΔR/R_0_ response to breathing.

Taking advantage of the sensitive resistance response of the ZHS to external strain, the self-powered sensing properties were systematically evaluated. As shown in Figure 4a, with an increase in strain, the open circuit voltage delivered by the ZHS remained constant at about 1.1 V, providing a stable output voltage for potential application which require stretching and motion detection. The resistive sensing performance of the ZHS were studied by recording the output current and voltage changes of the device along with those of a fixed resistor after applying external strains (Figure 4b). By linear fitting the calculated ΔR/R0 vs. strain, the sensitivity factor (gauge factors, GF) of the device could be obtained. Here, ΔR is the change of resistance according to the expression ΔR = R_i_ − R_0_, where R_0_ is the initial resistance of the ZHS and Ri represents the dynamic resistance under stretching. As shown in Figure 4c, the GF values of the self-powered sensor were 1.4 and 2.4 in strain ranges of 50–250% and 300–500%, respectively.

The device can be attached to the human knee, fingers and chest for motion and breathing detection. Figure 4d,e show the ΔR/R0 changes following repeated bending of the finger and knee, respectively. In these cases, the amplitudes of ΔR/R_0_ for the finger (ΔR/R_0_ = 1.25) and knee (ΔR/R_0_ = 0.55) were great enough to provide clear and quick motion detection. Additionally, the device could also be used for human respiration monitoring. As shown in Figure 4e, the sensor was placed on a human chest wall muscle using tape. It was interesting to see clear ΔR/R_0_ waves during breath cycles. This was because the sensor was stretched during the respiration process, bringing about different ΔR/R_0_ ratios. Therefore, as a proof of concept, this self-powered sensor could be employed for various motion detection and monitoring applications, and the present research may inspire the development of multifunctional electronic devices.

It is noted that hydrogels themselves are a promising type of sensor material, which have been already employed in various sensor designs. Recently, Gu et al. designed a macroporous conductive hydrogel as a strain sensor; its performance was shown to be very stable after 1200 consecutive cycles under 50% strain [41]. Liu et al. prepared a multifunctional hydrogel using the dynamic borate bonds between PVA and borax. The proposed sensor based on this hydrogel was self-healing after damage and maintained its sensing capacity [42]. Furthermore, Pei et al. reported a cellulose nanocrystal (CNC) nanocomposite hydrogel with extreme toughness and strain sensitivity, whose mechanical strength could reach to 5.7 MPa [43]. AuNWs/PAAm hydrogel-based sensors have reached a sensitivities which make them capable of monitoring pulse fluctuations in the human body [44]. This progress highlights the versatility of hydrogels and their potential for use in advanced sensors, as well as energy storage devices, making the development of promising multifunctional devices practical.

## 4. Conclusions

In summary, a bifunctional ZHS having both supercapacitor and sensor capabilities was designed. The double-crosslinking design in the Zn-alginate/PAAm hydrogel enables a stretchability of 1500% and impressive mechanical properties, which confer excellent electrochemical properties and adaptabilities upon the fabricated ZHS in various working conditions such as impact, bending and high temperature. The ZHS exhibited an energy density of 0.32 mWh cm^−2^ at a power density of 0.34 mW cm^−2^, a capacitance retention of 90% after 1000 cycles, and 60% capacitance retention at 70 °C. In particular, taking advantage of the resistance variation due to external strain, the ZHS device showed sensitive and self-powered sensing capabilities for human joint motions and breathing monitoring. This work provides the basis for the design of function-integrated devices for compact sensing applications.

## Data Availability

The data that support the findings of this study are available from the corresponding author, [Z.Z.], upon reasonable request.

## Supplementary Materials

The following supporting information can be downloaded at: www.mdpi.com/article/10.3390/ma15051767/s1, Figure S1: Adhesion properties of Zn-alginate/PAAm hydrogel; Figure S2: (a) Load test of the Zn-alginate/PAAm hydrogel. (b) The hydrogel remains intact under 1500% stretching; Figure S3: Water retention capabilities of Zn-alginate/PAAm and Na-alginate/PAAm hydrogels; Figure S4: (a) SEM image of VN powders; (b) XRD patterns of VN powders; Figure S5: Nyquist plot of the ZHS using Zn-alginate/PAAM hydrogel as the solid electrolyte; Figure S6: The cyclic stability of the ZHS device; Figure S7: CV curves of ZHS at large sweep speeds; Figure S8: High-temperature performance of ZHS; Figure S9: Photograph of the ZHS put at the flame of alcohol blast burner that can still work normally for over 20 s; Figure S10: Photographs of ZHS powering a digital clock at different bending conditions; Figure S11: Flexibility test of ZHS device.
