# Supplementary material for: Flexible and Wearable Zinc-Ion Hybrid Supercapacitor Based on Double-Crosslinked Hydrogel for Self-Powered Sensor Application
Source: Materials (Basel). 2022 Feb 26;15(5):1767. doi: 10.3390/ma15051767 (PMC8911391; doi:10.3390/ma15051767)
Supplement: Supplementary file 1 [file materials-15-01767-s001.zip › materials-1606793-supplementary.pdf]

Supplementary Materials

Flexible and Wearable Zinc-Ion Hybrid Supercapacitor Based on Double-Crosslinked Hydrogel for Self-Powered Sensor Application

Xi Wen [1,†], Kang Jiang [2,†], Heng Zhang [2], Hua Huang [3], Linyu Yang [1,*], Zeyan Zhou [2,*] and Qunhong Weng [2,*]

[1] School of Physical Science and Technology, Xinjiang University, Urumqi 830046, China; www6231659@163.com
[2] College of Materials Science and Engineering, Hunan University, Changsha 110016, China; jiangkang2@hnu.edu.cn (K.J.); 15873400812@163.com (H.Z.)
[3] Xinjiang Lixin Energy Co., Ltd., Urumqi 830046, China; huahuag_888@163.com
* Correspondence: yanglinyu0222@sina.com (L.Y.); zhouzeyan@hnu.edu.cn (Z.Z.); wengqh@hnu.edu.cn (Q.W.)
† These authors contributed equally to this work.

The capacitances C_{device} (F) were calculated from discharging profiles (GCD) according to equation (1):

$$C_{device} = It/U \quad (1)$$

in which U(V) is the working voltage window; ν (V s^{-1}) is the scan rate; I(A) is the current. And areal capacitances C_A (F cm^{-2}) were calculated by the following equation:

$$C_A = C_{device}/A \quad (2)$$

in which A(cm^2) is the effective area of the device. The areal energy density E_A (Wh cm^{-2}) and power density P_A (W cm^{-2}) were calculated from equation (3) and equation (4), respectively:

$$E_A = 1/2 \times C_A \times U^2/3600 \quad (3)$$

$$P_A = 3600 \times E_A/t \quad (4)$$

where t(s) is the discharging time of the device.

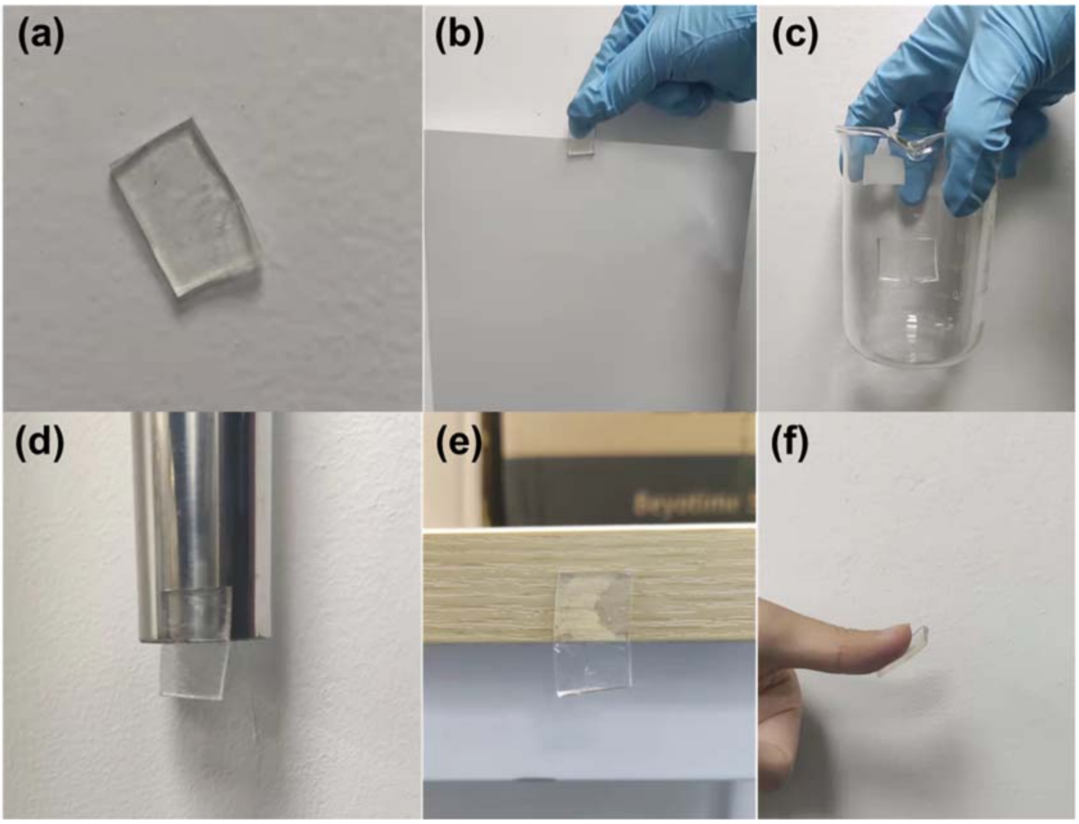

Figure S1. Adhesion properties of Zn-alginate/PAAm hydrogel. Photographs of the hydrogels attached (a) on a wall, (b) on a paper, (c) on a glass, (d) on a metal, (e) on a wood and (f) on skin.

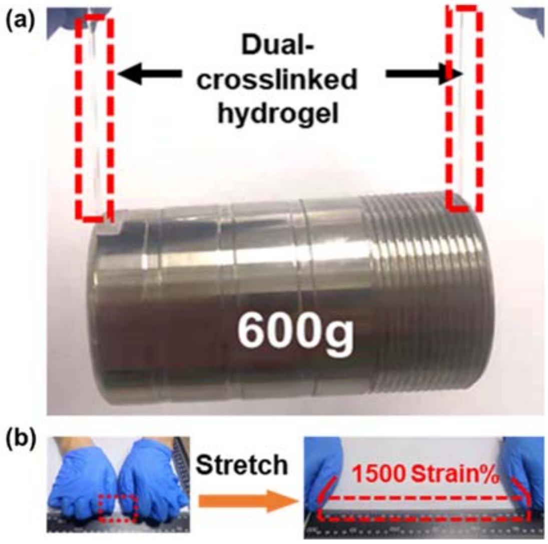

Figure S2. (a) Load test of the Zn-alginate/PAAm hydrogel. (b) The hydrogel remains intact under 1500% stretching.

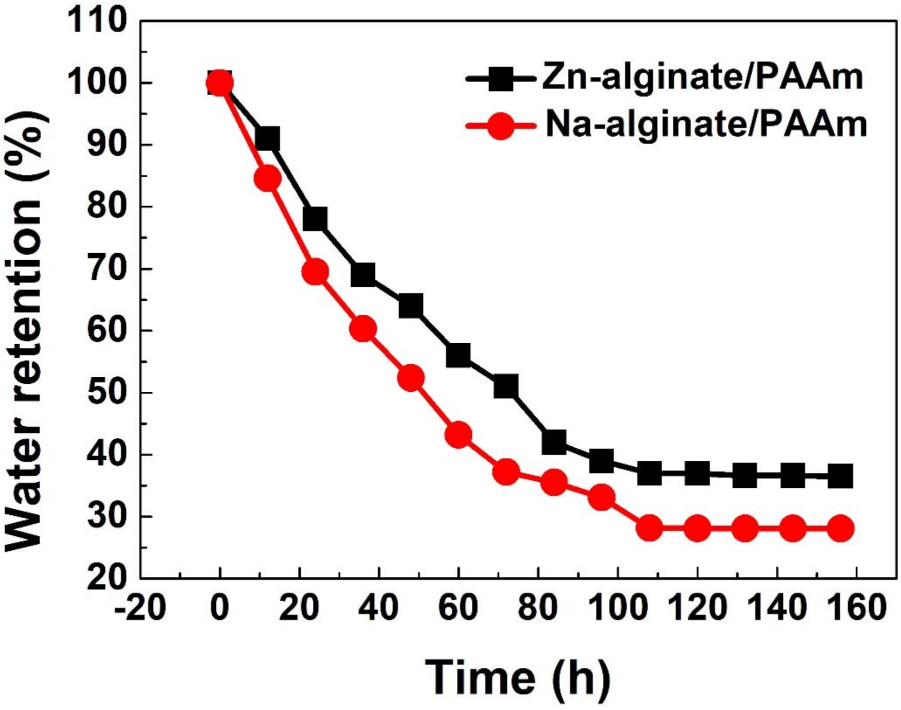

Figure S3. Water retention capabilities of Zn-alginate/PAAm and Na-alginate/PAAm hydrogels.

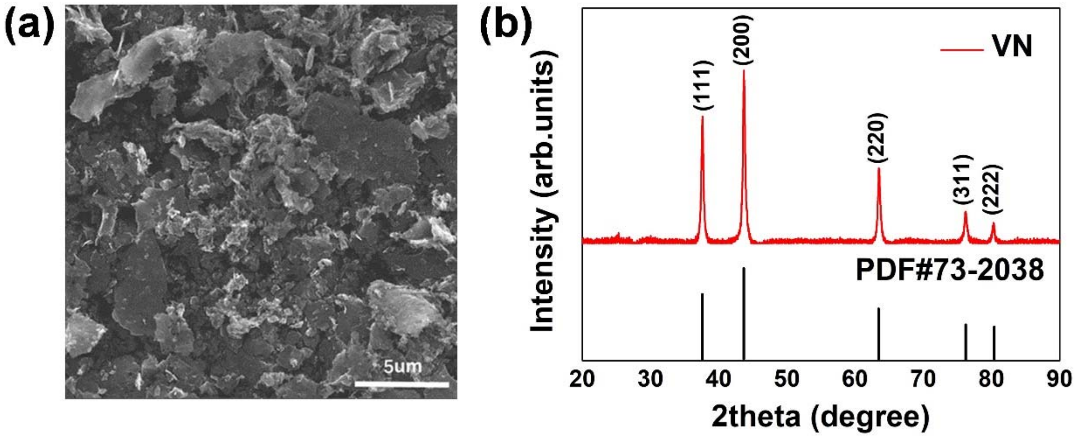

Figure S4. (a) SEM image of VN powders; (b) XRD patterns of VN powders.

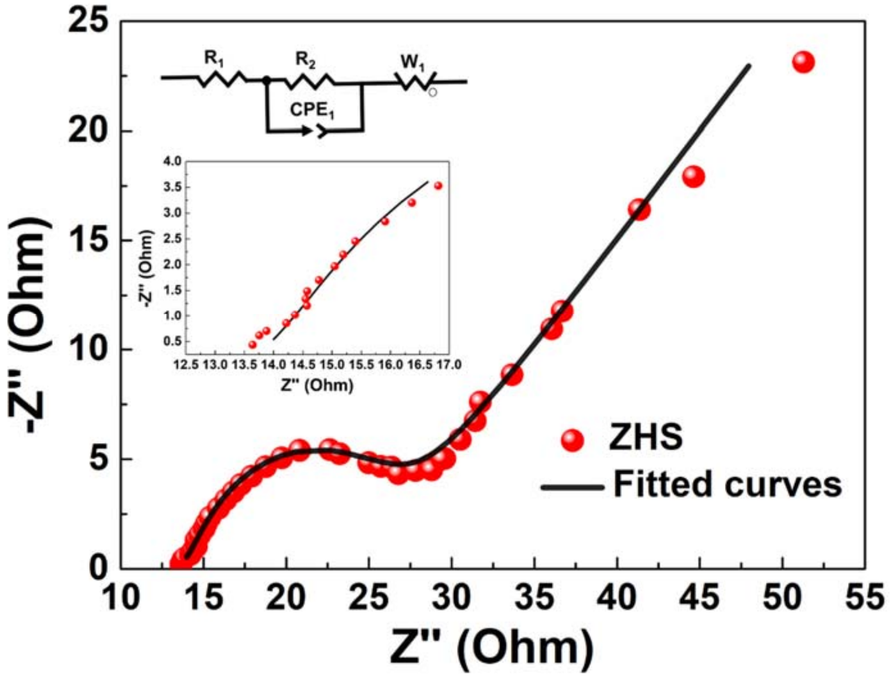

Figure S5. Nyquist plot of the ZHS using Zn-alginate/PAAM hydrogel as the solid electrolyte.

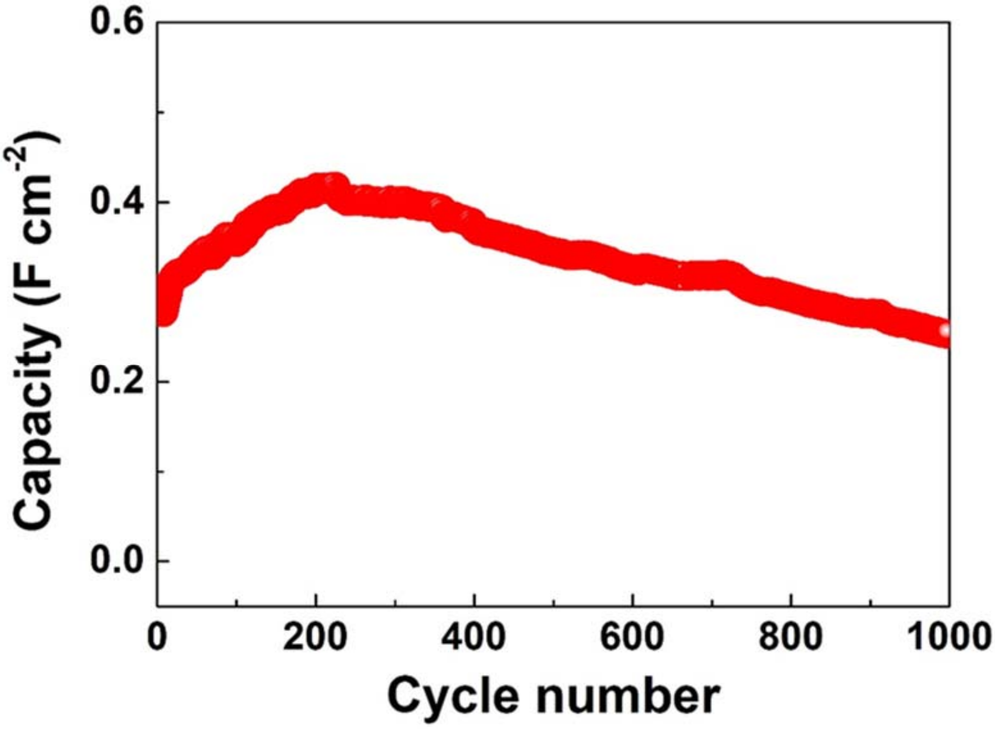

Figure S6. The cyclic stability of the ZHS device.

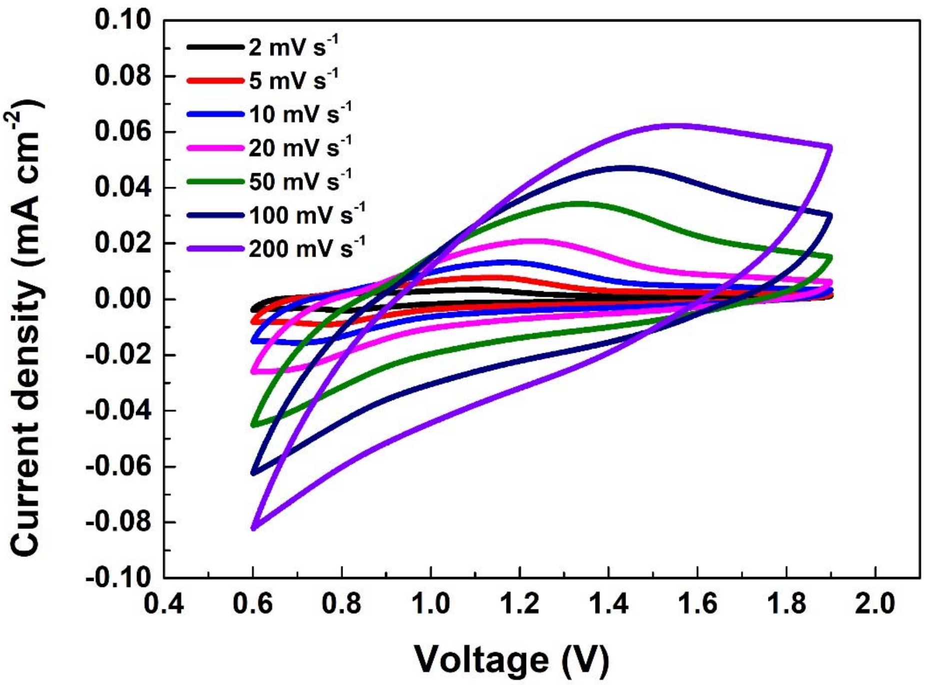

Figure S7. CV curves of ZHS at large sweep speeds.

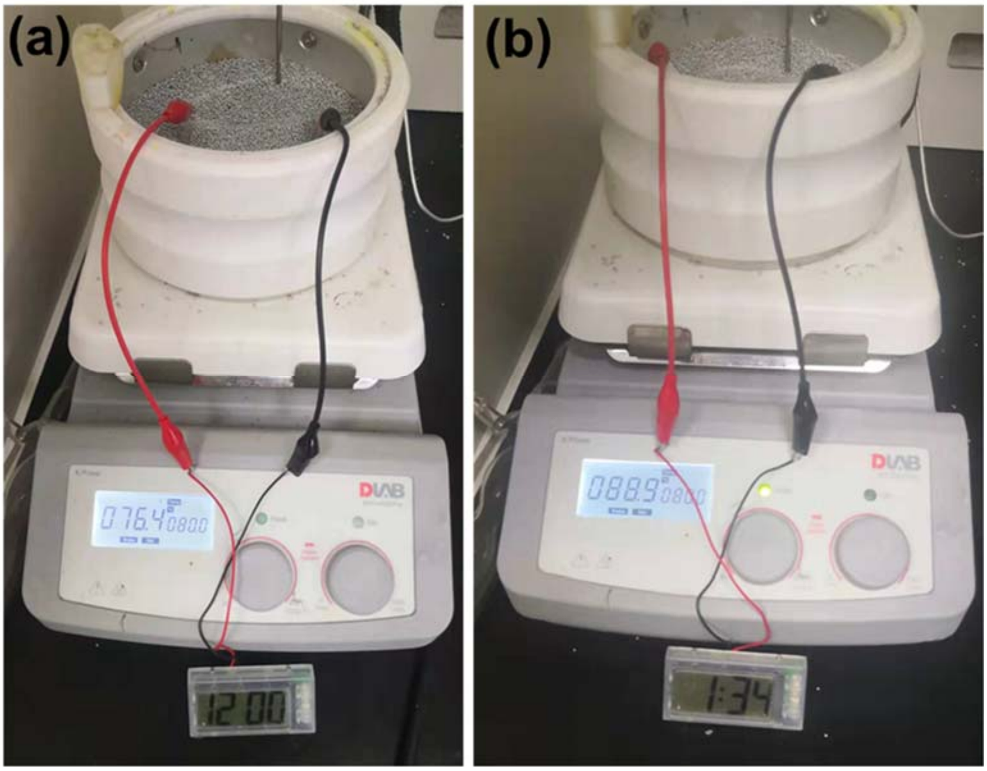

Figure S8. High-temperature performance of ZHS. (a) Photograph of ZHS buried in Al sand bath at 76 ^{o}C for powering a digital watch. (b) Photograph of ZHS buried in Al sand bath at 88 ^{o}C for powering a digital watch. The results show that the ZHS can still work normally at high temperatures.

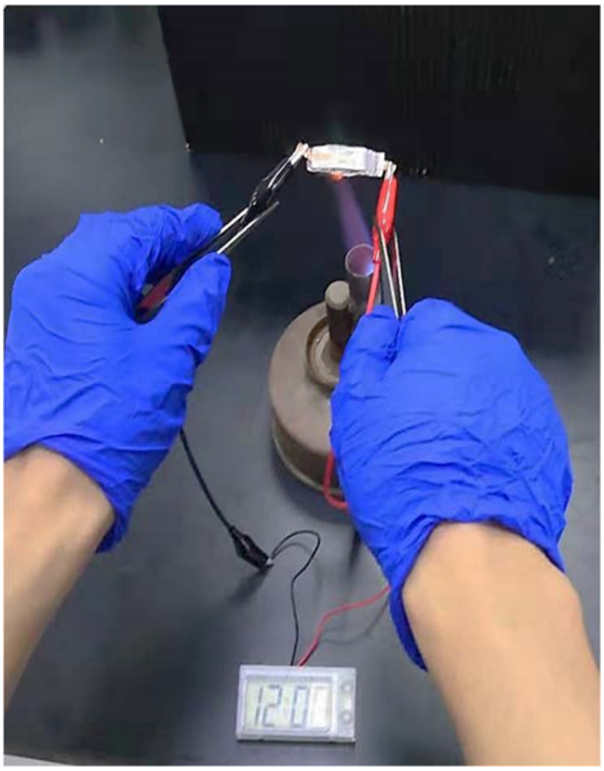

Figure S9. Photograph of the ZHS put at the flame of alcohol blast burner that can still work normally for over 20 seconds.

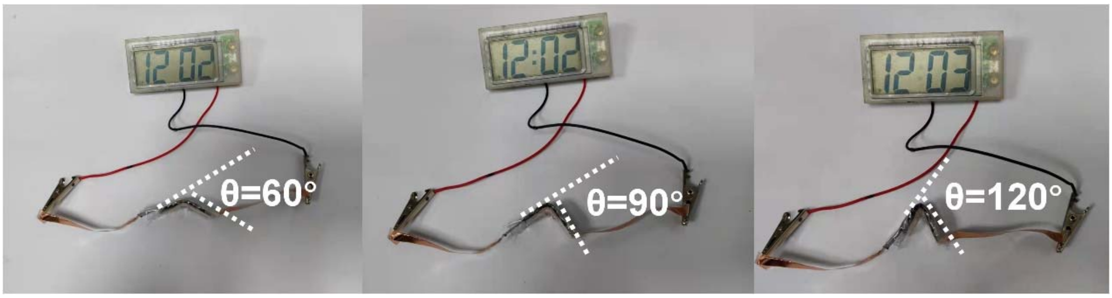

Figure S10. Photographs of ZHS powering a digital clock at different bending conditions.

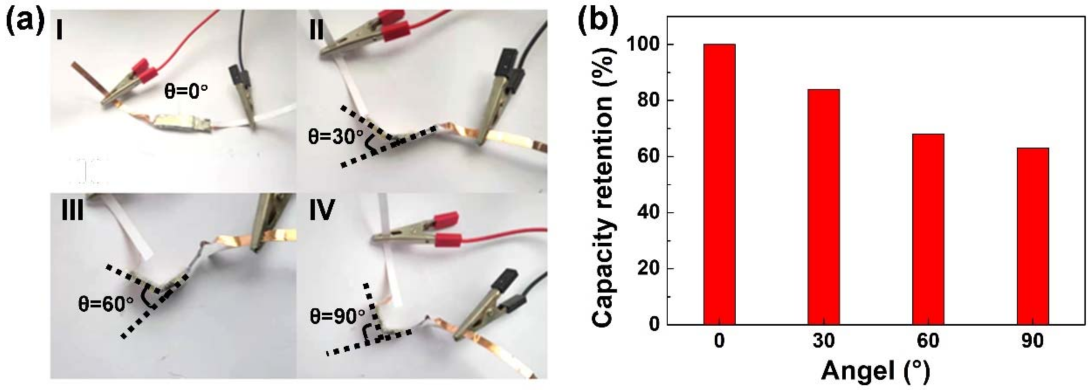

Figure S11. Flexibility test of ZHS device. (a) Device under different bending angles. (b) The recorded GCD curves of ZHS under different bending angles. The results show the ZHS can still work normally under the bending states.
